# Reasons for medical consultation among members of the Indian Scientific Expeditions to Antarctica

**DOI:** 10.3402/ijch.v72i0.20175

**Published:** 2013-02-14

**Authors:** Abhijeet Bhatia, Pradip Malhotra, Ashok Kumar Agarwal

**Affiliations:** National Centre for Antarctic and Ocean Research, Goa, India

**Keywords:** Maitri, India, Polar health

## Abstract

The article attempts to analyze the disease burden in a healthy, pre-screened population subjected to prolonged residence in the hostile environment of Antarctica. This retrospective epidemiological study was conducted utilizing data from medical consultation room on board the Indian Antarctic expedition vessels and at Indian Antarctic station, Maitri from seven Indian Scientific Expeditions to Antarctica (ISEA). The study group (n=327) consisted of 325 men and two women. The total number of medical room consultations was 1989. Maximum consultations were for injuries (27.25%); 14.68% were musculoskeletal and 10.31% were bruises and lacerations. Disturbances of gastrointestinal tract (19.66%) were the second most common disorders. Psychological disturbances accounted for 2.66% consultations. Cold injuries constituted 2.01% consultations and photophthalmia accounted for 1.06% consultations.

The Antarctic Treaty ensures that only scientific and other peaceful activities are conducted in Antarctica. Fifty nations are currently signatories to the treaty and there are 101 research stations, camps and refuges in Antarctica, some with year round activities and others seasonal (1). Due to the remoteness of the continent and the small size of national expeditions, extensive health care facilities are not feasible. Secondary referral and emergency evacuations are not always possible, and almost all eventualities have to be managed at the station. This article reports on the health problems encountered by members of the Indian Scientific Expeditions to Antarctica (ISEA), a generally healthy, pre-screened population who are subjected to prolonged residence in a hostile environment. It contributes to a limited pool of data on health conditions in Antarctica (2).

## Health care setting

India has a regular on-going scientific research program in Antarctica since 1981, when the first ISEA was launched (3). Each expedition consists of a summer and a winter team. The Indian Antarctic Station, Maitri (70°45′E, 11°44′S), is located in the Central Dronning Maudland region of east Antarctica, about 100 km inland from the Princess Astrid coast.

The expedition doctors provide health care in ISEA. No diagnostic or surgical facilities are available on board the expedition vessel, whereas Maitri has a consultation-treatment room, an operating theatre, a radiological unit and a basic laboratory for haematological and biochemical tests. Emergency air evacuation is not possible from March to November. Air service is available between November and February, linking Cape Town in South Africa and the Novo air base, which is approximately 10 km from Maitri.

The expedition team is selected from a pool of volunteers after interviews and detailed medical and psychological examinations. An acclimatisation and orientation camp at a high altitude location in the Indian Himalayas follows this.

## Sources of clinical data

Data were available for the 13th, 15th, 17th, 22nd, 24th, 27th and 29th ISEA, covering a period between 1993 and 2011. Two of the authors served as medical officers on the 22nd (Malhotra and Agarwal) and one on the 27th ISEA (Bhatia). The medical reports of various ISEA were obtained from the National Centre for Antarctic and Ocean Research (NCAOR), as well as by special requests to individual medical officers who served on the various expeditions. Expeditions for which the data was incomplete were excluded from the study. The health disorders in both the summer and the winter team of each expedition were recorded and tabulated. Foreign tourists, expeditioners and ship and helicopter crewmembers treated on board the expedition vessel or at Maitri by the ISEA doctors were also included in the analysis.

## Reasons for consultation

There were a total of 1,989 medical room consultations for 325 men and 2 women. Table I presents the number of medical room consultations by ISEA and also medical specialty. The reasons for consultation, all expeditions combined, classified according to the International Classification of Diseases (ICD-10), are shown in Table II.

**Table I:** Distribution of medical consultations by expedition and medical specialty

	13th ISEA	15th ISEA	17th ISEA	22nd ISEA	24th ISEA	27th ISEA	29th ISEA	Total	%
Medicine	245	78	426	55	53	232	66	1155	58.1
Surgery	120	64	20	26	42	37	9	318	16.0
Orthopaedics	101	16	171	19	0	25	7	339	17.0
Ophthalmology	14	1	13	6	5	11	2	52	2.6
Otolaryngology	34	11	0	4	0	18	7	74	3.7
Dentistry	14	15	0	6	9	2	5	51	2.6
	528	185	630	116	109	325	96	1989	100.0

**Table II:** Distribution of medical consultations by ICD-10 chapters

	ICD-10 chapters	*n*	%
	Total	1989	100.0
I	Certain infectious/parasitic	11	0.6
IV	Endocrine/nutritional/metabolic	18	0.9
V	Mental/behavioural	40	2.0
VI	Nervous	3	0.2
VII	Eye/adnexa	49	2.5
VIII	Ear/mastoid	21	1.1
IX	Circulatory	152	7.6
X	Respiratory	44	2.2
XI	Digestive	501	25.2
XII	Skin/subcutaneous	288	14.5
XIII	Musculoskeletal/connective	45	2.3
XIV	Genitourinary	6	0.3
XVIII	Symptoms/signs	217	10.9
XIX	Injury/poisoning	593	29.8
XXI	Contact with health system	1	0.1

Injuries were the most common reason for medical room presentation (30%), ranging from bruises and lacerations to fractures and dislocations. Injuries occurred more common in the convoy team because of heavy physical work in severe cold and rough terrain. Next commonest were conditions of the digestive tract such as diarrhoea, dyspepsia, and oral–dental conditions (25%). Psychological disturbances accounted for only 2% of consultations and included insomnia, anxiety and depression, likely the result of prolonged physical isolation. Neither cold injuries such as frostbite, chilblains and hypothermia (2%) nor “snow blindness” (1%) were common, likely due to the good quality clothing and protective eye gear available in ISEA. As with any primary care practice, skin conditions (15%) and signs, symptoms and ill-defined conditions (11%) are common.

## Discussion

As most Antarctic research programs conduct pre-induction health screening, the selected team members are generally healthy. Mortality and serious morbidity in Antarctica are mostly the result of accidents and hence potentially avoidable.

The importance of injuries to the health of people working in Antarctica has long been recognised (4–7). The prevalence of frostbite in ISEA is lower than that reported in a Japanese Antarctic Research Expedition (JARE) review (6). Among cold injuries occurring among members of the British Antarctic Survey between 1986 and 1995, 78% were reported as a consequence of outdoor recreational activities (8). Such activities are not encouraged by ISEA.

Psychological problems in Antarctica tend to appear in the winter during the third quarter of the isolation period (third quarter phenomenon) (9). They are rare in Maitri, perhaps because it has good recreation facilities to keep the ISEA team members occupied. These include facilities for indoor and outdoor games, books and video library, a multi-religion place of worship, and regular television shows. Internet and television broadcasts have been introduced since 2009.
